# Whole-Genome Sequencing-Based Species Classification, Multilocus Sequence Typing, and Antimicrobial Resistance Mechanism Analysis of the Enterobacter cloacae Complex in Southern China

**DOI:** 10.1128/spectrum.02160-22

**Published:** 2022-11-09

**Authors:** Xu Dong, Mei Zhu, Yaxuan Li, Dawei Huang, Lan Wang, Chunxia Yan, Linhua Zhang, Fubo Dong, Junwan Lu, Xi Lin, Kewei Li, Qiyu Bao, Cheng Cong, Wei Pan

**Affiliations:** a School of Medicine, Lishui University, Lishui, Zhejiang, China; b School of Laboratory Medicine and Life Sciences, Wenzhou Medical University, Wenzhou, Zhejiang, China; c Department of Clinical Laboratory, Zhejiang Hospital, Hangzhou, Zhejiang, China; d Medical Molecular Biology Laboratory, School of Medicine, Jinhua Polytechnic, Jinhua, Zhejiang, China; e The People’s Hospital of Yuhuan, Yuhuan, Zhejiang, China; Louis Stokes Cleveland Veteran’s Affairs Medical Center

**Keywords:** *Enterobacter cloacae* complex, whole-genome sequencing-based species classification, MLST, antimicrobial resistance mechanism, Pan-genome

## Abstract

Members of the Enterobacter cloacae complex (ECC) are important opportunistic nosocomial pathogens that are associated with a great variety of infections. Due to limited data on the genome-based classification of species and investigation of resistance mechanisms, in this work, we collected 172 clinical ECC isolates between 2019 and 2020 from three hospitals in Zhejiang, China and performed a retrospective whole-genome sequencing to analyze their population structure and drug resistance mechanisms. Of the 172 ECC isolates, 160 belonged to 9 classified species, and 12 belonged to unclassified species based on ANI analysis. Most isolates belonged to E. hormaechei (45.14%) followed by E. kobei (13.71%), which contained 126 STs, including 62 novel STs, as determined by multilocus sequence typing (MLST) analysis. Pan-genome analysis of the two ECC species showed that they have an “open” tendency, which indicated that their Pan-genome increased considerably with the addition of new genomes. A total of 80 resistance genes associated with 11 antimicrobial agent categories were identified in the genomes of all the isolates. The most prevailing resistance genes (12/29, 41.38%) were related to β-lactams followed by aminoglycosides. A total of 247 β-lactamase genes were identified, of which the *bla*_ACT_ genes were the most dominant (145/247, 58.70%), followed by the *bla*_TEM_ genes (21/247, 8.50%). The inherent ACT type β-lactamase genes differed among different species. *bla*_ACT-2_ and *bla*_ACT-3_ were only present in E. asburiae, while *bla*_ACT-9_, *bla*_ACT-12_, and *bla*_ACT-6_ exclusively appeared in E. kobei, E. ludwigii, and E. mori. Among the six carbapenemase-encoding genes (*bla*_NDM-1_, *bla*_NDM-5_, *bla*_IMP-1_, *bla*_IMP-4_, *bla*_IMP-26_, and *bla*_KPC-2_) identified, two (*bla*_NDM-1_ and *bla*_IMP-1_) were identified in an ST78 E. hormaechei isolate. Comparative genomic analysis of the carbapenemase gene-related sequences was performed, and the corresponding genetic structure of these resistance genes was analyzed. Genome-wide molecular characterization of the ECC population and resistance mechanism would offer valuable insights into the effective management of ECC infection in clinical settings.

**IMPORTANCE** The presence and emergence of multiple species/subspecies of ECC have led to diversity and complications at the taxonomic level, which impedes our further understanding of the epidemiology and clinical significance of species/subspecies of ECC. Accurate identification of ECC species is extremely important. Also, it is of great importance to study the carbapenem-resistant genes in ECC and to further understand the mechanism of horizontal transfer of the resistance genes by analyzing the surrounding environment around the genes. The occurrence of ECC carrying two MBL genes also indicates that the selection pressure of bacteria is further increased, suggesting that we need to pay special attention to the emergence of such bacteria in the clinic.

## INTRODUCTION

The Enterobacter cloacae complex (ECC) is one of the most common nosocomial pathogens causing healthcare-associated infections involving pneumonia, urinary tract infections, and septicemia (1). Previous studies have reported that the E. cloacae complex mainly comprises six species, including E. cloacae, Enterobacter asburiae, Enterobacter hormaechei, Enterobacter kobei, Enterobacter ludwigii, and Enterobacter nimipressuralis (2). Among them, E. hormaechei and E. cloacae are most frequently isolated from human clinical specimens (3). Based on the rapid development of next-generation sequencing, the most widely used method of species identification, *hsp60* typing, could be replaced by comparison of the sequenced Enterobacter genomes through whole-genome sequencing (WGS), which provides higher resolution to distinguish the E. cloacae complex at the taxonomic level. The ECC has been further classified into clades (A to V), including the Hoffmann cluster (I to XII) (4, 5).

Most isolates of the ECC produce the chromosomally encoded AmpC β-lactamase and intrinsically exhibit resistance to several antimicrobial agents, such as ampicillin, amoxicillin, amoxicillin-clavulanic acid, and first- and second-generation cephalosporins, whereas they are generally susceptible to fluoroquinolones, chloramphenicol, aminoglycosides, tetracyclines, trimethoprim-sulfamethoxazole, piperacillin-tazobactam, and carbapenems (2). Due to the selective pressure of antimicrobials in the clinic, an increasing number of ECC isolates carrying different acquired resistance genes have been detected. The ECC has become the third major drug-resistant *Enterobacteriaceae* species associated with nosocomial infections following Escherichia coli and Klebsiella pneumoniae due to the prevalence of Extended spectrum β-lactamases (ESBLs) and carbapenemases in the constituent species (6). The most clinically prevalent ESBLs are TEM, SHV, and CTX-M enzymes (7), and the major types of carbapenemases are KPC, NDM, and IMP/VIM (8). In China, the first reported carbapenemase in *Enterobacteriaceae* was KPC-2, identified in Shanghai in 2007 (9), and more types of carbapenemases (IMP, VIM, and NDM) have been identified in different geographical regions (10–12). The acquisition of these genes is most often associated with mobile genetic elements (MGEs), such as plasmids and transposons, that can be easily transferred between different species.

In this work, we performed a retrospective whole-genome sequencing to analyze the population structure and the distribution of resistance genes, especially focusing on resistance genes more concerned in the clinic (ESBLs and carbapenemases), among 172 clinical E. cloacae complex isolates collected between 2019 and 2020 from Zhejiang, China. We observed and identified a novel isolate that harbored two metallobeta-lactamases (MBLs).

## RESULTS

### Characteristics of clinical ECC isolates.

**FIG 1:**
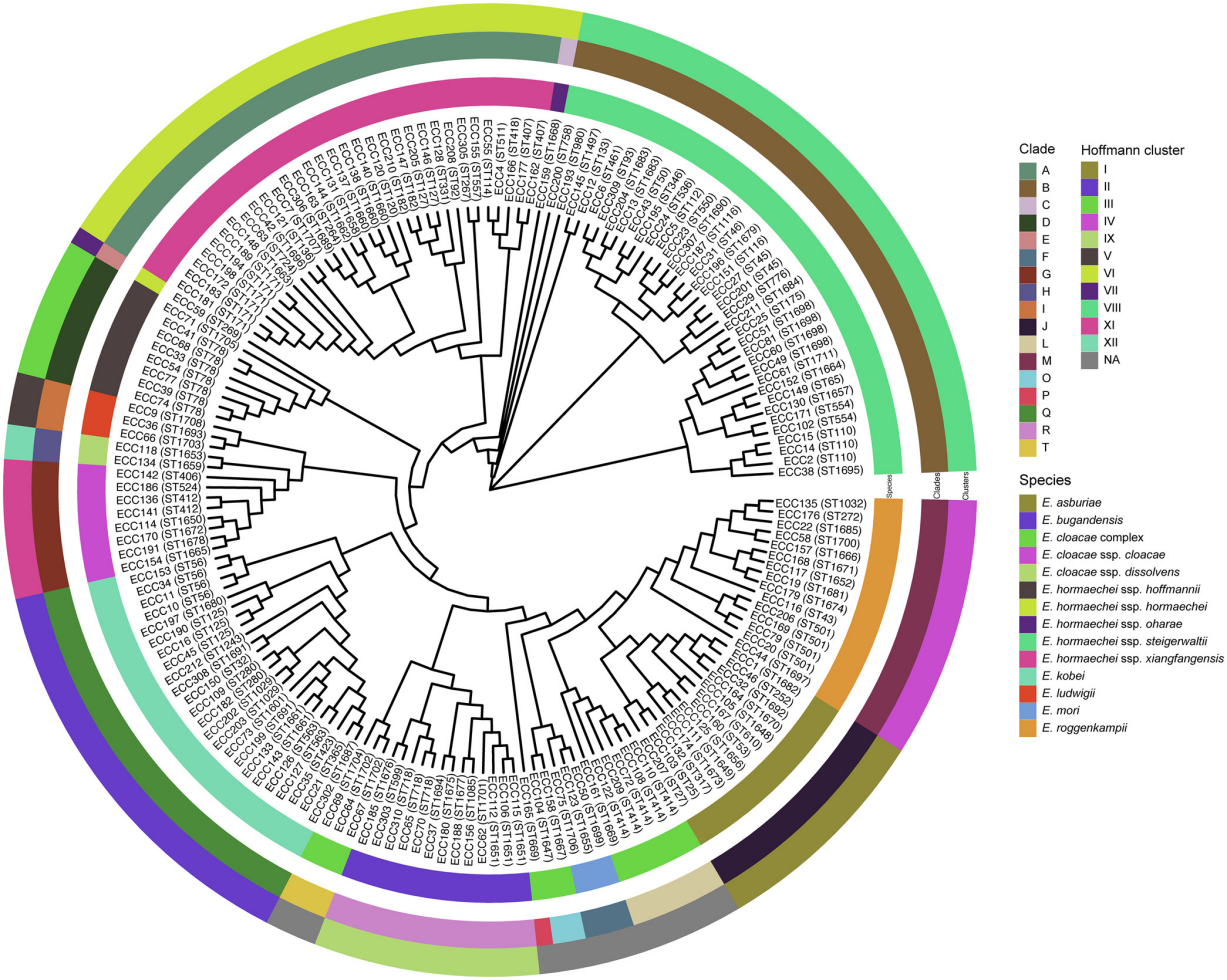
SNP phylogenetic tree of the genomes of 172 E. cloacae complex isolates. The species/subspecies, clades, and Hoffmann clusters are drawn in concentric circles.

A total of 172 ECC strains were isolated from different sources: wound secretion (*n* = 55), sputum (*n* = 46), urine (*n* = 21), throat swab (*n* = 13), blood (*n* = 11), pus (*n* = 10), bile (*n* = 5), ascites (*n* = 3), bronchoalveolar lavage (*n* = 2), tissue (*n* = 2), drainage (*n* = 2), catheter (*n* = 1), and semen (*n* = 1). The average nucleotide identity (ANI) results of 172 isolates revealed that the predominant species was E. hormaechei (*n* = 79) followed by E. kobei (*n* = 24), E. roggenkampii (*n* = 15), E. asburiae (*n* = 14), E. bugandensis (*n* = 13), E. cloacae (*n* = 10), E. ludwigii (*n* = 3), and E. mori (*n* = 3). Twelve isolates with ANI values below the threshold (95%) for the classified species/subspecies were grouped into 4 clades (L, O, P, and T) (Table 1; Fig. 1). These 172 ECC isolates exhibited high susceptibility to amikacin (98.8%), gentamicin (93.6%), meropenem (90.1%), ciprofloxacin (78.5%), cefepime (77.3%), tigecycline (76.7%), ceftazidime (71.5%), chloramphenicol (68.6%), fosfomycin (59.9%), and tetracycline (54.7%) and low susceptibility to colistin (28.0%), ampicillin (0.6%), and cefoxitin (0%) (Table 2). Ten isolates (5.8%, 10/172) exhibited an ESBL-positive phenotype, most of which belonged to E. hormaechei (*n* = 6), followed by E. kobei (*n* = 2). Among the 14 isolates with carbapenem resistance phenotypes, E. hormaechei was the predominant species (*n* = 9) followed by E. kobei (*n* = 3).

**TABLE 1:** ECC isolates used in this study

Strain	Species	Clade	hsp60 typing	ST	Location
1	E. asburiae	J	I	1682-NEW	Hangzhou
2	E. hormaechei subsp. steigerwaltii	B	VIII	110	Hangzhou
4	E. hormaechei subsp. xiangfangensis	A	VI	511	Hangzhou
5	E. hormaechei subsp. steigerwaltii	B	VIII	112	Hangzhou
6	E. hormaechei subsp. steigerwaltii	B	VIII	461	Hangzhou
7	E. hormaechei subsp. xiangfangensis	A	VI	1707-NEW	Hangzhou
9	E. ludwigii	I	V	1708-NEW	Hangzhou
10	E. kobei	Q	II	56	Hangzhou
11	E. kobei	Q	II	56	Hangzhou
12	E. hormaechei subsp. steigerwaltii	B	VIII	133	Hangzhou
13	E. hormaechei subsp. steigerwaltii	B	VIII	1683-NEW	Hangzhou
14	E. hormaechei subsp. steigerwaltii	B	VIII	110	Hangzhou
15	E. hormaechei subsp. steigerwaltii	B	VIII	110	Hangzhou
16	E. kobei	Q	II	125	Hangzhou
19	E. roggenkampii	M	IV	1681-NEW	Hangzhou
20	E. roggenkampii	M	IV	501	Hangzhou
21	E. kobei	Q	II	365	Hangzhou
22	E. roggenkampii	M	IV	1685-NEW	Hangzhou
23	E. hormaechei subsp. steigerwaltii	B	VIII	550	Hangzhou
24	E. hormaechei subsp. steigerwaltii	B	VIII	536	Hangzhou
25	E. hormaechei subsp. steigerwaltii	B	VIII	175	Hangzhou
27	E. hormaechei subsp. steigerwaltii	B	VIII	45	Hangzhou
29	E. hormaechei subsp. steigerwaltii	B	VIII	776	Hangzhou
31	E. hormaechei subsp. steigerwaltii	B	VIII	46	Hangzhou
32	E. asburiae	J	I	1692-NEW	Hangzhou
33	E. hormaechei subsp. hoffmannii	D	III	78	Hangzhou
34	E. kobei	Q	II	56	Hangzhou
35	E. kobei	Q	II	423	Hangzhou
36	E. ludwigii	I	V	1693-NEW	Hangzhou
37	E. bugandensis	R	IX	1694-NEW	Hangzhou
38	E. hormaechei subsp. steigerwaltii	B	VIII	1695-NEW	Hangzhou
39	E. hormaechei subsp. hoffmannii	D	III	78	Hangzhou
41	E. hormaechei subsp. hoffmannii	D	III	78	Hangzhou
42	E. hormaechei subsp. xiangfangensis	A	VI	1696-NEW	Hangzhou
43	E. hormaechei subsp. steigerwaltii	B	VIII	50	Hangzhou
44	E. roggenkampii	M	IV	1697-NEW	Hangzhou
45	E. kobei	Q	II	125	Hangzhou
46	E. asburiae	J	I	252	Hangzhou
49	E. hormaechei subsp. steigerwaltii	B	VIII	1698-NEW	Hangzhou
50	E. mori	F		1699-NEW	Hangzhou
51	E. hormaechei subsp. steigerwaltii	B	VIII	1698-NEW	Hangzhou
54	E. hormaechei subsp. hoffmannii	D	III	78	Hangzhou
55	E. hormaechei subsp. xiangfangensis	A	VI	114	Hangzhou
58	E. roggenkampii	M	IV	1700-NEW	Hangzhou
59	E. hormaechei subsp. hormaechei	E	VII	269	Hangzhou
60	E. hormaechei subsp. steigerwaltii	B	VIII	1698-NEW	Hangzhou
61	E. hormaechei subsp. steigerwaltii	B	VIII	1711-NEW	Hangzhou
62	E. bugandensis	R	IX	1701-NEW	Hangzhou
63	E. hormaechei subsp. xiangfangensis	A	VI	724	Hangzhou
64	E. cloacae complex	T		1702-NEW	Hangzhou
65	E. bugandensis	R	IX	718	Hangzhou
66	E. ludwigii	I	V	1703-NEW	Hangzhou
67	E. cloacae complex	T		1702-NEW	Hangzhou
68	E. hormaechei subsp. hoffmannii	D	III	78	Hangzhou
69	E. cloacae complex	T		1704-NEW	Hangzhou
70	E. bugandensis	R	IX	718	Hangzhou
71	E. hormaechei subsp. hoffmannii	D	III	1705-NEW	Hangzhou
72	E. cloacae complex	L		414	Hangzhou
73	E. kobei	Q	II	1601-NEW	Hangzhou
74	E. hormaechei subsp. hoffmannii	D	III	78	Hangzhou
75	E. mori	F		1706-NEW	Hangzhou
77	E. hormaechei subsp. hoffmannii	D	III	78	Hangzhou
79	E. roggenkampii	M	IV	501	Hangzhou
81	E. hormaechei subsp. steigerwaltii	B	VIII	1698-NEW	Hangzhou
102	E. hormaechei subsp. steigerwaltii	B	VIII	554	Wenzhou
103	E. asburiae	J	I	25	Wenzhou
104	E. cloacae complex	O		1647-NEW	Wenzhou
105	E. asburiae	J	I	1648-NEW	Wenzhou
106	E. bugandensis	R	IX	1651-NEW	Wenzhou
108	E. cloacae complex	L		414	Wenzhou
109	E. kobei	Q	II	280	Wenzhou
110	E. cloacae complex	L		414	Wenzhou
111	E. asburiae	J	I	1649-NEW	Wenzhou
112	E. bugandensis	R	IX	1651-NEW	Wenzhou
114	E. cloacae subsp. cloacae	G	XI	1650-NEW	Wenzhou
115	E. bugandensis	R	IX	1651-NEW	Wenzhou
116	E. roggenkampii	M	IV	43	Wenzhou
117	E. roggenkampii	M	IV	1652-NEW	Wenzhou
118	E. cloacae subsp. dissolvens	H	XII	1653-NEW	Wenzhou
120	E. hormaechei subsp. xiangfangensis	A	VI	120	Wenzhou
121	E. hormaechei subsp. xiangfangensis	A	VI	136	Wenzhou
122	E. cloacae complex	L		414	Wenzhou
123	E. mori	F		1655-NEW	Wenzhou
125	E. asburiae	J	I	1656-NEW	Wenzhou
126	E. kobei	Q	II	563	Wenzhou
127	E. kobei	Q	II	563	Wenzhou
128	E. hormaechei subsp. xiangfangensis	A	VI	331	Wenzhou
130	E. hormaechei subsp. steigerwaltii	B	VIII	1657-NEW	Wenzhou
131	E. hormaechei subsp. xiangfangensis	A	VI	1658-NEW	Wenzhou
132	E. asburiae	J	I	317	Wenzhou
133	E. kobei	Q	II	1661-NEW	Wenzhou
134	E. cloacae subsp. dissolvens	H	XII	1659-NEW	Wenzhou
135	E. roggenkampii	M	IV	1032	Wenzhou
136	E. cloacae subsp. cloacae	G	XI	412	Wenzhou
137	E. hormaechei subsp. xiangfangensis	A	VI	1660-NEW	Wenzhou
138	E. hormaechei subsp. xiangfangensis	A	VI	1660-NEW	Wenzhou
140	E. hormaechei subsp. xiangfangensis	A	VI	1660-NEW	Wenzhou
141	E. cloacae subsp. cloacae	G	XI	412	Wenzhou
142	E. cloacae subsp. cloacae	G	XI	406	Wenzhou
143	E. kobei	Q	II	1661-NEW	Wenzhou
144	E. hormaechei subsp. xiangfangensis	A	VI	1662-NEW	Wenzhou
145	E. hormaechei subsp. steigerwaltii	B	VIII	1497-NEW	Wenzhou
146	E. hormaechei subsp. xiangfangensis	A	VI	127	Wenzhou
147	E. hormaechei subsp. xiangfangensis	A	VI	182	Wenzhou
148	E. hormaechei subsp. xiangfangensis	A	VI	1663-NEW	Wenzhou
149	E. hormaechei subsp. steigerwaltii	B	VIII	65	Wenzhou
150	E. kobei	Q	II	32	Wenzhou
151	E. hormaechei subsp. steigerwaltii	B	VIII	116	Wenzhou
152	E. hormaechei subsp. steigerwaltii	B	VIII	1664-NEW	Wenzhou
153	E. kobei	Q	II	56	Wenzhou
154	E. cloacae subsp. cloacae	G	XI	1665-NEW	Wenzhou
155	E. hormaechei subsp. xiangfangensis	A	VI	557	Wenzhou
156	E. bugandensis	R	IX	1085	Wenzhou
157	E. roggenkampii	M	IV	1666-NEW	Wenzhou
158	E. cloacae complex	O		1667-NEW	Wenzhou
159	E. hormaechei subsp. oharae	C	VI	1668-NEW	Wenzhou
160	E. asburiae	J	I	53	Wenzhou
161	E. cloacae complex	L		1669-NEW	Wenzhou
162	E. hormaechei subsp. xiangfangensis	A	VI	407	Wenzhou
163	E. hormaechei subsp. xiangfangensis	A	VI	264	Wenzhou
164	E. asburiae	J	I	1670-NEW	Wenzhou
165	E. cloacae complex	P		669	Wenzhou
166	E. hormaechei subsp. xiangfangensis	A	VI	418	Wenzhou
167	E. asburiae	J	I	610	Wenzhou
168	E. roggenkampii	M	IV	1671-NEW	Wenzhou
169	E. roggenkampii	M	IV	501	Wenzhou
170	E. cloacae subsp. cloacae	G	XI	1672-NEW	Wenzhou
171	E. hormaechei subsp. steigerwaltii	B	VIII	554	Wenzhou
172	E. hormaechei subsp. xiangfangensis	A	VI	171	Wenzhou
174	E. asburiae	J	I	1673-NEW	Wenzhou
176	E. roggenkampii	M	IV	272	Wenzhou
177	E. hormaechei subsp. xiangfangensis	A	VI	407	Wenzhou
179	E. roggenkampii	M	IV	1674-NEW	Wenzhou
180	E. bugandensis	R	IX	1675-NEW	Wenzhou
181	E. hormaechei subsp. xiangfangensis	A	VI	171	Wenzhou
182	E. kobei	Q	II	280	Wenzhou
183	E. hormaechei subsp. xiangfangensis	A	VI	171	Wenzhou
185	E. bugandensis	R	IX	1676-NEW	Wenzhou
186	E. cloacae subsp. cloacae	G	XI	524	Wenzhou
187	E. hormaechei subsp. steigerwaltii	B	VIII	1116	Wenzhou
188	E. bugandensis	R	IX	1677-NEW	Wenzhou
189	E. hormaechei subsp. xiangfangensis	A	VI	171	Wenzhou
190	E. kobei	Q	II	125	Wenzhou
191	E. cloacae subsp. cloacae	G	XI	1678-NEW	Wenzhou
193	E. hormaechei subsp. steigerwaltii	B	VIII	980	Wenzhou
194	E. hormaechei subsp. xiangfangensis	A	VI	171	Wenzhou
195	E. hormaechei subsp. steigerwaltii	B	VIII	346	Wenzhou
196	E. hormaechei subsp. steigerwaltii	B	VIII	1679-NEW	Wenzhou
197	E. kobei	Q	II	1680-NEW	Wenzhou
198	E. hormaechei subsp. xiangfangensis	A	VI	171	Wenzhou
199	E. kobei	Q	II	691	Wenzhou
200	E. hormaechei subsp. steigerwaltii	B	VIII	758	Wenzhou
201	E. hormaechei subsp. steigerwaltii	B	VIII	45	Wenzhou
202	E. kobei	Q	II	1029	Wenzhou
203	E. kobei	Q	II	1029	Wenzhou
204	E. hormaechei subsp. steigerwaltii	B	VIII	1683-NEW	Wenzhou
205	E. hormaechei subsp. xiangfangensis	A	VI	127	Wenzhou
206	E. roggenkampii	M	IV	501	Wenzhou
207	E. asburiae	J	I	27	Wenzhou
208	E. hormaechei subsp. xiangfangensis	A	VI	92	Wenzhou
209	E. cloacae complex	L		414	Wenzhou
210	E. hormaechei subsp. xiangfangensis	A	VI	182	Wenzhou
211	E. hormaechei subsp. steigerwaltii	B	VIII	1684-NEW	Wenzhou
212	E. kobei	Q	II	1243	Wenzhou
302	E. kobei	Q	II	1687-NEW	Huzhou
303	E. bugandensis	R	IX	599	Huzhou
305	E. hormaechei subsp. xiangfangensis	A	VI	267	Huzhou
306	E. hormaechei subsp. xiangfangensis	A	VI	1689-NEW	Huzhou
307	E. hormaechei subsp. steigerwaltii	B	VIII	1690-NEW	Huzhou
308	E. kobei	Q	II	1691-NEW	Huzhou
309	E. hormaechei subsp. steigerwaltii	B	VIII	93	Huzhou
310	E. bugandensis	R	IX	718	Huzhou

**TABLE 2:** Susceptibility profiles and MICs for 172 ECC strains

Antibiotics	% Resistant	% Intermediate	% Susceptible	MIC_50_ (mg/L)	MIC_90_ (mg/L)	MIC range (mg/L)
Ampicillin	95.9	3.5	0.6	256	>1024	1-1024
Cefoxitin	99.4	0.6	0.0	512	1024	0.5-1024
Ceftazidime	25.0	3.5	71.5	0.25	256	0.125-256
Cefepime	16.3	6.4	77.3	0.25	32	0.125-256
Meropenem	8.1	1.7	90.1	0.125	0.5	0.0125-64
Gentamicin	6.4	0.0	93.6	0.25	2	0.125-256
Amikacin	1.2	0.0	98.8	1	4	0.5-512
Ciprofloxacin	14.5	7.0	78.5	0.125	2	0.125-256
Chloramphenicol	16.3	15.1	68.6	8	256	1-1024
Fosfomycin	22.1	18.0	59.9	64	256	1-1024
Colistin	71.5	28.5	0.0	16	256	0.125-256
Tigecycline	3.5	19.8	76.7	1	4	0.125-256
Tetracycline	18.0	27.3	54.7	4	64	1-256

### Distribution of resistance genes among ECC isolates.

A total of 80 antibiotic resistance genes (>80 similarity with the function characterized resistance genes) associated with 11 antimicrobial agent categories were identified among the genomes of all the isolates, such as aminoglycosides (*aph(6)-Id*, *aac(6′)-IIc*, *aac(6′)-Ib*, *aph(3′’)-Ib*, and *aac(3)-Iia*), beta-lactam (*bla*_NDM-1_, *bla*_KPC-2_, *bla*_OXA-1_, *bla*_CTX-M-15_, *bla*_IMP-1_, *bla*_DHA-1_, and *bla*_SHV-12_), fluoroquinolone (*qnrE1* and *qnrS2*), fosfomycin (*fosA3*), macrolide (*ereA*), phenicol (*catA2* and *floR*), polymyxin (*mcr-10*), rifampicin (*arr-6*), sulfonamide (*sul2*), tetracycline (*tetA*), and trimethoprim (*dfrA12*) (Fig. S1 in Supplemental File 1). The most prevalent resistance gene type belonged to β-lactams (30/80, 37.50%) followed by aminoglycosides (17/80, 21.25%).

**FIG 2:**
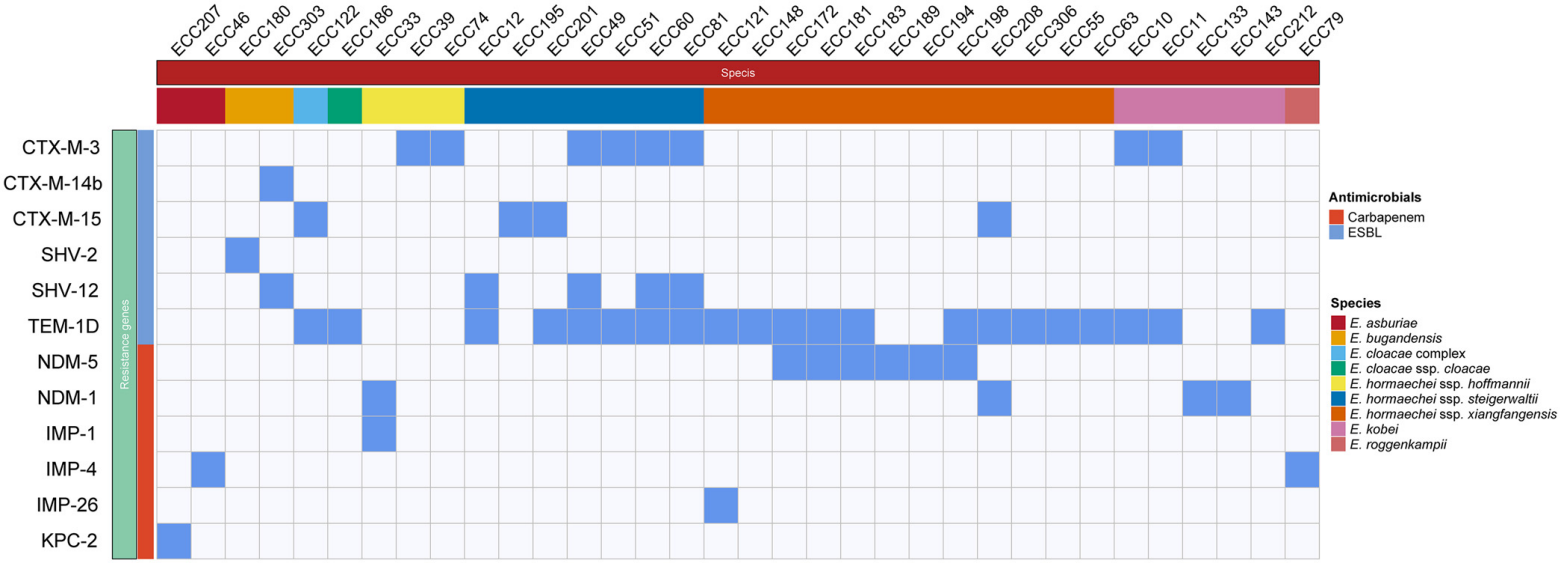
Distributions of acquired β-lactamase genes in the 26 isolates. Blue and white squares represent the presence and absence of genes, respectively.

A large number (247) of β-lactamase genes were identified in the 172 genomes. The *bla*_ACT_ genes were the most dominant (145/247, 58.70%) followed by the *bla*_TEM_ genes (21/247, 8.50%, *bla*_TEM-1D_ only), while the *bla*_KPC_ genes (1/247, 0.40%, *bla*_KPC-2_) had the lowest frequency. *fosA* genes were found in most strains (153/172, 88.95%), covering all species except E. hormaechei subsp. hormaechei, E. hormaechei subsp. oharae, and E. ludwigii (Fig. S1 in Supplemental File 1). Given the widespread use of β-lactam antibiotics in clinical settings, in this work, we gave more attention to resistance genes encoding beta-lactamases, especially those acquired horizontally (Fig. 2). As shown in Fig. S1 in Supplemental File 1, the distribution of some inherent AmpCs differed among different species. For example, *bla*_ACT-2_ and *bla*_ACT-3_ were only present in E. asburiae, whereas *bla*_ACT-9_, *bla*_ACT-12_, and *bla*_ACT-6_ were exclusively identified in E. kobei, E. ludwigii, and E. mori. Of note, ESBL genes and MBL genes were predominant among the horizontally acquired genes. ESBL genes were found in 26 isolates, with *bla*_TEM-1D_ being the most prevalent (*n* = 18) followed by *bla*_CTX-M-3_ (*n* = 8), and *bla*_SHV-12_ (*n* = 5). Carbapenem-resistant genotypes were present in 14 strains, with *bla*_NDM-5_ being the most prevalent (*n* = 6) followed by *bla*_NDM-1_ (*n* = 4), *bla*_IMP-4_ (*n* = 2), *bla*_IMP-1_ (*n* = 1), *bla*_IMP-26_ (*n* = 1), and *bla*_KPC-2_ (*n* = 1). It was also interesting that among isolates carrying the ESBLs or carbapenem genes, only *bla*_NDM-5_ showed some species specificity, appearing only in E. hormaechei subsp. xiangfangensis. Importantly, one isolate belonging to the E. hormaechei subsp. hoffmannii harbored two MBL genes, *bla*_NDM-1_ and *bla*_IMP-1_.

### MLST analysis.

MLST analysis showed that these 172 ECC isolates were divided into 126 STs, including 62 novel STs (ST1497, ST1601, ST1647-1653, ST1655-1685, ST1687, ST1689-1708, and ST1711) (Table 1). Among these STs, the predominant epidemic type was ST78 (*n* = 7), followed by ST171 (*n* = 6) and ST414 (*n* = 5) (Table 1; Fig. S2 in Supplemental File 1).

### Pan-genome analysis of E. hormaechei and E. kobei.

Because of the high frequency of the acquired resistance genes in the two species E. hormaechei and E. kobei, a pan-genome analysis was performed to determine the dynamics of the bacterial genomes. The results showed that a total of 2,932 core genes, 7,879 accessory genes (genes present in two or more genomes), and 8,629 unique genes (a gene specific to a single genome (13)) were found in the E. hormaechei strains. Similarly, there were 3,603 core genes, 3,577 accessory genes, and 4,216 unique genes among the E. kobei strains (Fig. S3 in Supplemental File 1).

The rarefaction curve of the two species revealed that as genomes were sampled, the number of pan-genome genes did not show a trend of saturation, which indicated that the pan-genome of the two species were open according to the Heaps’ law model. The tendency for an open status, to some extent, meant that species with a higher number of pangenome genes had a greater capacity to acquire exogenous genes. However, E. hormaechei showed a lower number of core genes but a higher number of accessory and unique genes than E. kobei, which might be due to the diversity of E. hormaechei strains, which included more (5) subspecies.

To further understand the functional differences of pangenome genes, Cluster of Orthologous Groups of proteins (COG) functional classification of core genes, accessory genes, and unique genes of the two species was performed. The results indicated that the two species shared a highly similar COG category repartition of the core genomes, mainly associated with the functional classification of inorganic ion transport and metabolism, transcription, amino acid transport, and metabolism. Compared with the core genome, the unique and accessory genomes presented greater abundance in replication, recombination, and repair, which indicated that the functions of these genes are more likely to be associated with plasmid maintenance (14). Interestingly, there was a higher proportion of genes with unknown function (35.57% to 36.01%), indicating that the pan-genome genes of the two species have not been intensively studied yet.

### Genetic environment of carbapenem-resistant genes.

Six carbapenemase genes were identified in 14 ECC isolates, including *bla*_NDM-1_, *bla*_NDM-5_, *bla*_IMP-1_, *bla*_IMP-4_, *bla*_IMP-26_, and *bla*_KPC-2_. As shown in Fig. S4A in Supplemental File 1, the genetic context of *bla*_KPC-2_ in the IncR plasmid pECC207-88 was consistent with that of another Enterobacter cloacae IncR plasmid, pHN84KPC (KY296104). However, the transposase of the Tn*3* unit upstream of *bla*_KPC-2_ differed from the classical Tn*1722*-*bla*_KPC-2_-unit transposon in the Klebsiella pneumoniae IncR plasmid pKP048 (FJ628167). Although the flanks of *bla*_KPC-2_ were enwrapped by IS*Kpn6* and IS*Kpn27* in both pKP048 and pECC207-88, the transposon followed by IS*Kpn27* in pECC207-88 was an insertion sequence that shared 84% nucleotide similarity with IS*Ec63*, a 4,473 bp element that belonged to the Tn*3* family. Notably, similar IS*Ec63*-like elements have also been reported in other Klebsiella pneumoniae strains with nonclassical transposon elements (15).

The genetic environments surrounding *bla*_NDM-1_ are shown in Fig. S4B in Supplemental File 1. Four plasmids carrying *bla*_NDM-1_ could be classified into different sequence types. The type A plasmid lacked the IS*Aba125* element upstream of IS*5*, and the genes downstream of *groEL* were different from those in the previously reported IncX3 plasmid pNDM-ECN49 (KP765744). The *cutA1* gene of the type B plasmid was truncated by the insertion of IS*26*. Thus, the genes downstream of *cutA1* changed greatly compared with the sequences of the type A plasmid. The transposase also exhibited a difference between IS*3000* and ΔIS*Aba125*. Furthermore, similar to the type A plasmid, the sequence structure of type B lacked the IS*Aba125* element upstream of IS*1* compared with the epidemic IncX3 plasmid pZHH-4 (CP059715). The genetic structure of the type C plasmid shared the highest similarity with the transposon Tn*6460*, which was composed of Tn*6292* and a truncated Tn*3000*. However, compared with Tn*6460*, Tn*6292* was inserted into the middle region of ΔTn*3000* (*dsbD* gene) instead of the region downstream of ΔTn*3000*. Notably, no structure completely consistent with that of the type C plasmid was found in the NCBI nucleotide database. Six plasmids possessed the same genetic background of *bla*_NDM-5_ (IS*3000*-IS*Aba125*-IS*5*-ΔIS*Aba125*-*bla*_NDM-5_-*ble*_MBL_-*trpF*-*dsbC*-Δ*cutA1*-IS*26*-*umuD*-IS*Kox3*), which is the same as that of IncX3 plasmid pNDM_MGR194 (KF220657) from Klebsiella pneumoniae strain MGR-K194 isolated in India (Fig. S4C in Supplemental File 1).

All of the IMP genes were located in class 1 integrons (Fig. S4D in Supplemental File 1). The gene cassette of the *bla*_IMP-26_-containing integron was *bla*_IMP-26_-*ltrA*-*qacE*Δ*1*-*sul1*, which was the same as that of IncHI2 plasmid pIMP26 from E. cloacae (MH399264). Two IncN plasmids (pECC46-54 and pECC79-52) carrying *bla*_IMP-4_ had a structurally similar genetic context except for the insertion of an IS*Sen4* between *bla*_IMP-4_ and *ltrA*. Moreover, the genes surrounding *bla*_IMP-4_ in these two plasmids were identical to IncN plasmids pIMP-HK1500 (KT989599) and pP10159-2 (MF072962), both from Citrobacter freundii. The *bla*_IMP-1_ gene in pECC33-49 was situated within a Tn*402*-like integron, which was atypical due to a lack of 3′-CS. A similar genetic structure of *bla*_IMP-1_ (*intI1-aacA7-*Δ*orfE-bla*_IMP-1_*-ΔtniA*) was also found in IncP-1β plasmid pA22732-IMP (KJ588780) from Achromobacter xylosoxidans. However, the *orfE*-like gene was truncated to only 64 bp in length.

### Identification and characterization of an NDM-1- and IMP-1-coproducing ECC ECC33.

The isolate ECC33, harboring two MBLs (NDM-1- and IMP-1) and conferring high-level resistance to meropenem (MIC of 256 μg/mL), was isolated from the urine of an old patient who was diagnosed with urinary tract infection in a tertiary hospital in Zhejiang, China. The ANI result revealed that ECC33 shared the highest identity (99.05%) with E. hormaechei subsp. hormaechei ATCC 49162 (AFHR00000000). MLST showed that ECC33 belonged to ST78, which was thought to be a high-risk clone among both ESBL-producing ECC and carbapenem-resistant Enterobacter cloacae complex (CREC) (16). The ECC33 genome consisted of a circular chromosome and two plasmids. The *bla*_IMP-1_ was carried on the IncP-1β plasmid pECC33-49, which encoded 56 open reading frames (ORFs) with a length of 49,381 bp, and the *bla*_NDM-1_ was carried on the IncN plasmid pECC33-57, which was 57,389 bp in length and contained 72 ORFs.

**FIG 3:**
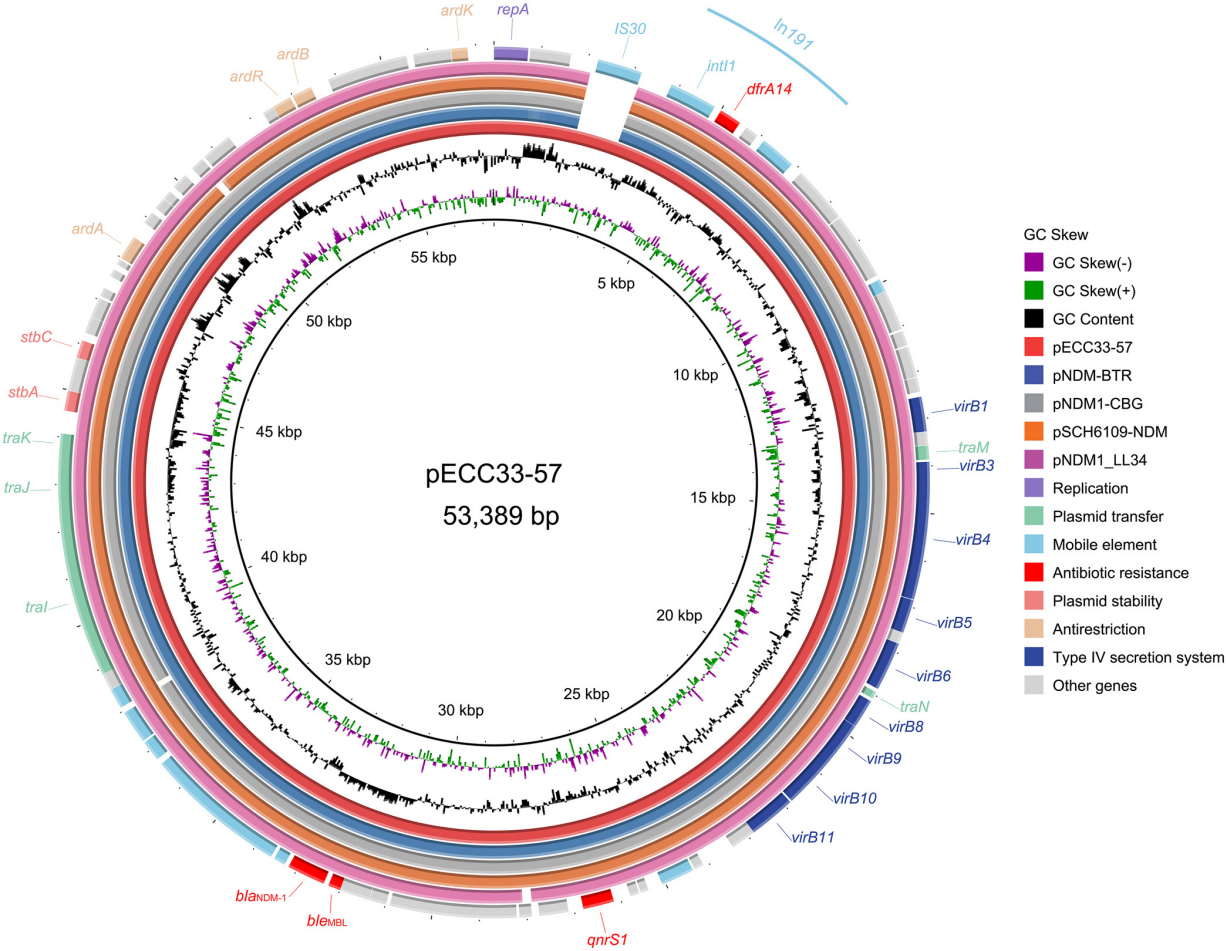
Genomic comparison of the plasmid pECC33-57 (red inner circle) with its close relatives. The ORFs of different gene functions are denoted by rectangles in various colors. Genes present in pECC33-57 but absent in the other plasmids are shown as blank spaces in the rings.

pECC33-57 carried several antimicrobial resistance genes (*bla*_NDM-1_, *ble*, *qnrS1*, and *dfrA14*) conferring resistance to β-lactams, bleomycin, quinolones, and trimethoprim. Sequence analysis indicated that pECC33-57 also carried different mobile genetic elements (MGEs), including one class 1 integron In*191* (Fig. 3). Comparative genomic analysis revealed that pECC33-57 shared the highest sequence similarities with four *bla*_NDM-1_-carrying IncN plasmids, namely, pNDM1-CBG (CP046118.1; 97% coverage and 100% identity), pSCH6109-NDM (CP050859.2; 97% coverage and 99.95% identity), pNDM1_LL34 (CP025965.2; 97% coverage and 99.99% identity) and pNDM-BTR (KF534788.2; 98% coverage and 99.99% identity), especially true in the classical and highly conserved backbones of these plasmids (Fig. 3). Interestingly, compared with these four plasmids, one extra IS*30* inserted upstream of In*191* was observed in pECC33-57, which may be associated with the mobilization of In*191*.

**FIG 4:**
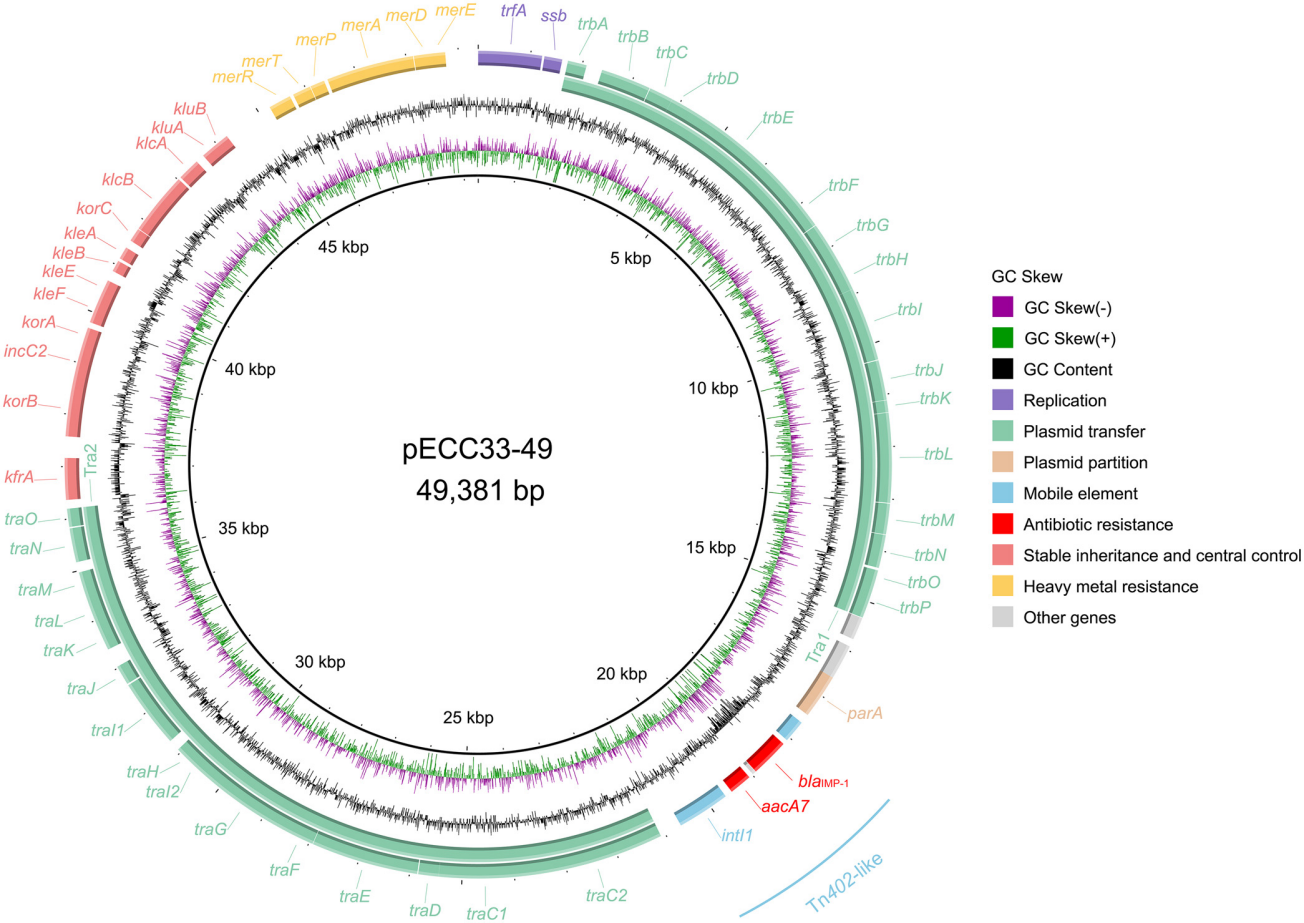
Schematic map of pECC33-49. The ORFs are colored based on gene function classification. The circles represent (from outside to inside) the predicted coding sequences (CDS); GC content; GC skew; and scale in kilobase pairs.

Only one antimicrobial resistance gene, *bla*_IMP-1_, located within a Tn*402*-like integron, was found in pECC33-49. An operon related to resistance to mercury (*merEDAPTR*) was also found in pECC33-49. Moreover, plasmid pECC33-49 contained two transfer-related regions, one consisting of 15 *tra* genes (*traC* to *traO*) and the other consisting of 16 *trb* genes (*trbA* to *trbP*) (Fig. 4). Notably, when searching for pECC33-49-like genomes (>80% coverage and >80 identity) in the NCBI database, none of the related genomes identified were from the Enterobacter cloacae complex. In contrast, we found four *bla*_IMP-1_-encoding IncP-1β plasmids from Achromobacter xylosoxidans (pA22732-IMP, 99.95% coverage and 99.98% identity; KJ588780.1), Morganella morganii (pNXM63-IMP, 98.76% coverage and 99.99% identity; MW150990.1), and Aeromonas caviae (pKAM345_1, 91.60% coverage and 99.93% identity; AP024949.1; pKAM339_2, 91.61% coverage and 99.93% identity; AP024942.1). Phylogenetic analysis of these five IncP-1β plasmids revealed that pECC33-49 has a close relationship with plasmid pNXM63-IMP (MW150990.1) from Morganella morganii (Fig. S5 in Supplemental File 1). This finding indicated that the pECC33-49-like plasmids were more likely to be transferred between bacterial species of different genera.

## DISCUSSION

ECC are common nosocomial pathogens ranking as the top three *Enterobacteriaceae* in healthcare-associated infections (3), which has attracted wide public attention. Nevertheless, the presence and emergence of multiple species/subspecies of ECC have led to diversity and complications at the taxonomic level. Moreover, it impedes the further understanding of the epidemiology and clinical significance of species/subspecies of ECC. Accurate identification of ECC species is extremely important. Previous studies have mainly classified ECC species by phenotype or *hsp60* typing (2). With the rapid development of sequencing technology, genotypic classification methods, such as ANI, have higher accuracy and resolution. In this work, 172 ECC clinical isolates were collected from three hospitals in different cities in Zhejiang Province in southern China, and then further classified 160 into 9 species based on ANI analysis. E. hormaechei (45.14%) and E. kobei (13.71%) were the predominant species, and the high prevalence of E. hormaechei and E. kobei among ECC isolates was consistent with the findings of a previous study that investigated the characterization of ESBL-positive community-acquired ECC isolates from 31 hospitals in 12 provinces of China (6). Interestingly, because novel species recently described based on a computational analysis of sequenced Enterobacter genomes (4, 17), E. bugandensis and E. roggenkampii were also identified in this study, accounting for 7.56% and 8.72% of the isolates, respectively. However, due to the limited sequencing data available in public databases, 12 isolates without characterized species/subspecies references could only be clustered into 4 clades (L, O, P and T) according to the classification method described by Sutton et al. (4), and these isolates will be further analyzed and might be classified as novel species/subspecies.

One hundred and twenty-six STs, including 62 novel STs, were found among the 172 isolates, with ST78 (4.07%) and ST171 (3.49%) ranking first and second, respectively. This large number and a great variety of STs, especially with half of the STs in such a small population being novel, indicated the members of the ECC group might have been evolving in clinical settings. A similar phenomenon was observed in a multicenter study in which novel STs accounted for a significant proportion (87.50%) (18). ST78 and ST171 were the most prevalent STs among the isolates in this work. A previous study revealed that ST78 and ST171, as high-risk CREC clones, were widely distributed and had high epidemic potential (19). The potential drug-resistant outbreaks caused by these STs still need robust surveillance.

Almost all of the isolates (170/172; 98.86%) carried AmpC β-lactamase genes, with *bla*_ACT_ genes being the most dominant (58.70%), indicating that inducible ACT AmpC enzymes are conserved among ECCs. Intriguingly, the ACT type β-lactamase genes exhibited a certain species specificity. For instance, *bla*_ACT-2_ and *bla*_ACT-3_ were only present in E. asburiae, whereas *bla*_ACT-9_, *bla*_ACT-12_, and *bla*_ACT-6_ exclusively occurred in E. kobei, E. ludwigii, and E. mori, respectively. DHA, the most prevalent plasmid-mediated AmpC β-lactamase that might confer slight resistance to carbapenem (20), was found to be less abundant in the E. cloacae complex. Interestingly, compared to the previous findings that the *bla*_NDM-1_ gene was most prevalent among the carbapenem-resistant isolates found in northeastern (e.g., Liaoning Province) (21), southern (e.g., Guangdong Province), and northwestern (e.g., Ningxia) (22) regions, the CREC strains we found here were dominated by *bla*_NDM-5_ (6/14; 42.86%), which might be due to differences in antibacterial agents use in different regions of China, or may be caused by the limited number of strains we collected. Moreover, a higher frequency of other acquired β-lactamase genes, such as *bla*_TEM,_ was observed in E. hormaechei and E. kobei isolates, indicating that the strains of the two species are more likely to spread and survive in hospital settings. Pan-genome analysis of the two species revealed that they possessed a greater capacity to acquire exogenous genes (23), which might partly explain the higher frequency of resistance genes in these species.

Notably, a strain named ECC33 harboring two MBL genes, *bla*_NDM-1_ and *bla*_IMP-1_, was identified in our study. To the best of our knowledge, although a previous study has reported an NDM-1 and IMP-1 co-expressing E. cloacae strain based on next-generation sequencing in Ningxia in northwest China (22), this is the first report of detailed characterization of NDM-1 and IMP-1 encoded on two plasmids, respectively, in an E. hormaechei isolate. MLST assigned this isolate to ST78, a high-risk clone among both ESBL-producing ECC and CREC (19). There is no doubt that the emergence of the ST78 clone harboring two MBLs increases the difficulty of clinical treatment. Interestingly, compared with the *bla*_IMP-1_-encoding plasmid pECC33-49 from ECC33, 4 similar plasmid sequences (from Achromobacter xylosoxidans, Morganella morganii, and Aeromonas caviae) with >91.0% coverage and >99.0% identity were found from genera other than the E. cloacae complex in the NCBI database. The diversity of the origins indicated that the pECC33-49-like plasmids are widely distributed in different species and may be captured by E. hormaechei through the high-frequency transfer of the recombinant plasmids.

## MATERIALS AND METHODS

### Clinical strain collection.

A large number of isolates from patients with active disease were continuously collected from three districts (Wenzhou, Hangzhou, and Huzhou) in Zhejiang Province, China between 2019 and 2020. After genetic identification of isolates as strains of Enterobacter cloacae complex, a total of 172 clinical ECC isolates were obtained from three tertiary hospitals (100 strains from hospital A in Wenzhou, 64 strains from hospital B in Hangzhou, and 8 strains from hospital C in Huzhou) for further study.

### Antimicrobial susceptibility test.

Antimicrobial susceptibility was determined using the agar dilution method on Mueller-Hinton (MH) agar plates supplemented with different concentrations of antibiotics. The test was repeated three times to ensure accuracy. The MICs were then interpreted following the breakpoint criteria of the Clinical and Laboratory Standards Institute (CLSI) for Enterobacteriaceae (24). Escherichia coli ATCC 25922 was used as the MIC reference strain for quality control. Those isolates which conferred resistance to meropenem were considered to be carbapenem resistance phenotypes. ESBL confirmation test was also performed according to the method recommended by CLSI and positive strains were considered to be ESBL positive phenotype.

### Whole-genome sequencing and sequence analysis.

Genomic DNA was extracted from each isolate using an AxyPrep bacterial genomic DNA miniprep kit (Axygen Scientific, Union City, CA, USA). The library with an average insert size of 400 bp was prepared using NEBNext Ultra II DNA library preparation kit, and genomic sequencing by the Illumina HiSeq 2500 platform (paired-end run; 2 × 150 bp) was performed at Shanghai Sunny Biotechnology Co., Ltd. (Shanghai, China). Genomic sequencing was performed by the Illumina HiSeq 2500 platform. Sequence assembly was conducted *de novo* on Illumina short reads using SPAdes v.3.14.0 (25). The genomes of isolates carrying MBL genes were further sequenced by PacBio RS II instruments (Pacific Biosciences, CA, USA). The PacBio data were first assembled using Canu v2.1 (26) and then the assembled genomes were corrected using Illumina HiSeq data via Pilon v1.23 (27). Gene annotation was performed using the Prokka annotation pipeline (28) and corrected via BLASTN (https://blast.ncbi.nlm.nih.gov/Blast.cgi?PROGRAM=blastn&PAGE_TYPE=BlastSearch&LINK_LOC=blasthome). Antimicrobial resistance genes were detected using ResFinder (29), and plasmid replicon types were identified using PlasmidFinder (30). MLST analysis was performed using the MLST database (http://pubmlst.org/ecloacae). The population evolutionary relationship of ST was analyzed using GrapeTree (https://enterobase.readthedocs.io/en/latest/index.html). Mobile genetic elements (MGEs) were identified using ISFinder (31) and INTEGRALL (32) with default parameters. Gene distribution was visualized using the ComplexHeatmap package in R (33). Core genes of E. cloacae complex genomes were constructed using kSNP (34) to call single nucleotide polymorphisms (SNPs) and the recombined regions within the core genome were detected using Gubbins v2.4.1 (35). The phylogenetic tree was visualized in ggtree package in R (36). Genetic context analysis was performed using BLASTN and visualized in genoplotR (37). The BLAST Ring Image Generator (BRIG) (38) tool was used to generate the circular maps of the plasmids pECC33-57 and pECC33-49.

### Pangenome analysis and COG functional characterization.

Analysis of the pangenomes of two species (E. hormaechei and E. kobei) was carried out using Roary v3.13.0 (39). The rarefaction curve of pan-genomes and core-genomes of selected species was visualized via ggplot2 (40). COG categorization of each pan-genome was performed using EggNOG-mapper v 2.1.0 (41) with an E value < 1e−10, an identity higher than 40%, and a coverage higher than 70%.

### Species identification using ANI.

Due to the diversity of the subspecies of the Enterobacter cloacae complex, average nucleotide identity (ANI) analysis was performed for the isolates collected. The type strains used for the ANI analysis are listed in Table S1 in Supplemental File 1. The ANI analysis was computed using FastANI v1.31 (42), and a value >95% was used as the threshold for species definition.

### Ethics approval.

Individual patient data were not involved, and only anonymous clinical residual samples during routine hospital laboratory procedures were used in this study. It was approved by the ethics committee of Zhejiang Hospital, Zhejiang, China.

### Data availability.

The complete nucleotide sequences of the chromosome and two plasmids (pECC33-49 and pECC33-57) of ECC33 in this work have been submitted to the GenBank database under accession numbers CP098486, CP098487, and CP098488, respectively. The raw data of isolates collected in this study have also been submitted to the NCBI SRA database under the accession number PRJNA871306.
