# Supplementary material for: Whole-Genome Sequencing-Based Species Classification, Multilocus Sequence Typing, and Antimicrobial Resistance Mechanism Analysis of the Enterobacter cloacae Complex in Southern China
Source: Microbiol Spectr. 2022 Nov 9;10(6):e02160-22. doi: 10.1128/spectrum.02160-22 (PMC9769718; doi:10.1128/spectrum.02160-22)
Supplement: Supplemental file 1 — Supplemental material. Download spectrum.02160-22-s0001.pdf, PDF file, 3.2 MB [file spectrum.02160-22-s0001.pdf]

Table S1 reference genomes used for ANI in this study

subspecies	Strain	clade	Hoffmann cluster	BioSample ID or accession number
E. asburiae	ATCC 35953	J	I	NZ_CP011863.1
E. bugandensis	EB-247	R	IX	LT992502.1
E. cancerogenus	ATCC 33241	U		CP045769.1
E. cloacae complex clade K	1161_ECLO	K		SAMN03197118
E. cloacae complex clade L	GN02587	L		SAMN03732717
E. cloacae complex clade N	DS11005	N		SAMN07448201
E. cloacae complex clade O	GN05526	O		SAMN04578342
E. cloacae complex clade P	624_ECLO	P		SAMN03197824
E. cloacae complex clade S	ND22	S		SAMN05212257
E. cloacae complex clade T	C9	T		SAMN06237083
E. cloacae ssp. *cloacae*	ATCC 13047	G	XI	CP001918.1
E. cloacae ssp. *dissolvens*	SDM	H	XII	NC_018079.1
E. hormaechei ssp. *hoffmannii*	DSM 14563	D	III	NZ_CP017186.1
E. hormaechei ssp. *hormaechei*	ATCC 49162	E	VII	SAMN05787340
E. hormaechei ssp. *oharae*	DSM 16687	C	VI	NZ_CP017180.1
E. hormaechei ssp. *steigerwaltii*	DSM 16691	B	VIII	NZ_CP017179.1
E. hormaechei ssp. *xiangfangensis*	LMG27195	A	VI	NZ_CP017183.1
E. kobei	DSM 13645	Q	II	NZ_CP017181.1
E. ludwigii	EN-119	I	V	CP017279.1
E. mori	LMG 25706	F		SAMN02471025
E. roggenkampii	DSM 16690	M	IV	NZ_CP017184.1
E. soli	LMG 25861	V		SAMEA104113920

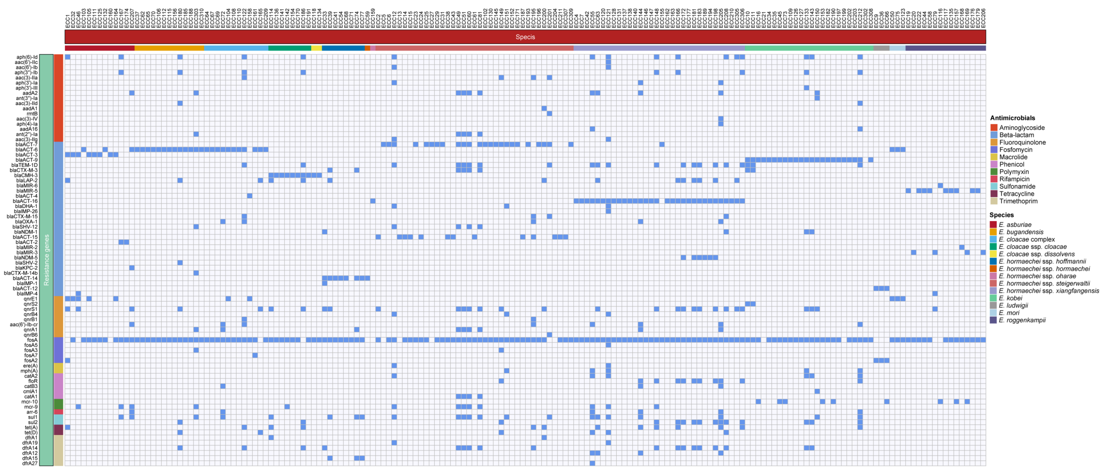

Figure S1. Distributions of resistance genes in the 172 isolates. Blue and white squares represent the presence and absence of genes, respectively. The categories of resistance genes and the species of these strains are marked with different colors.

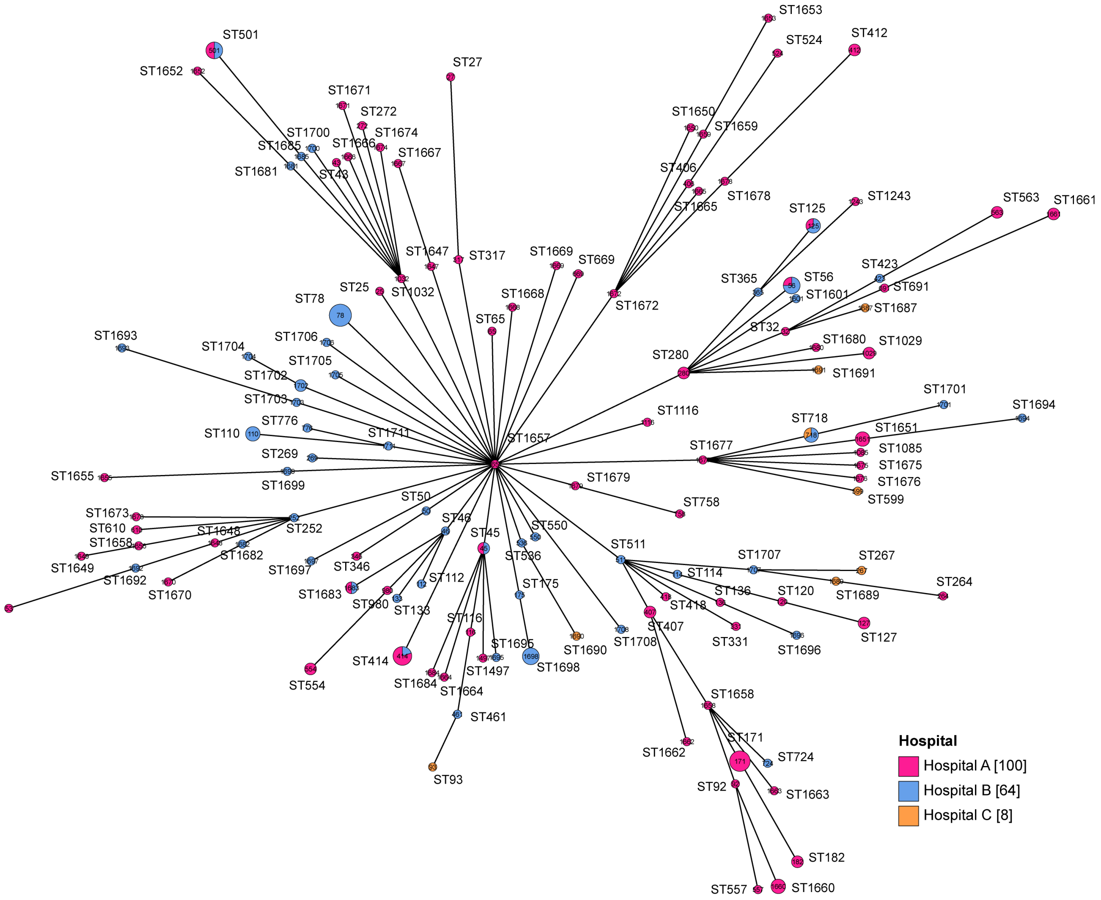

Figure S2. Population structure of the 172 ECC isolates. The number in each circle represents an ST. The size of the node represents the number of isolates.

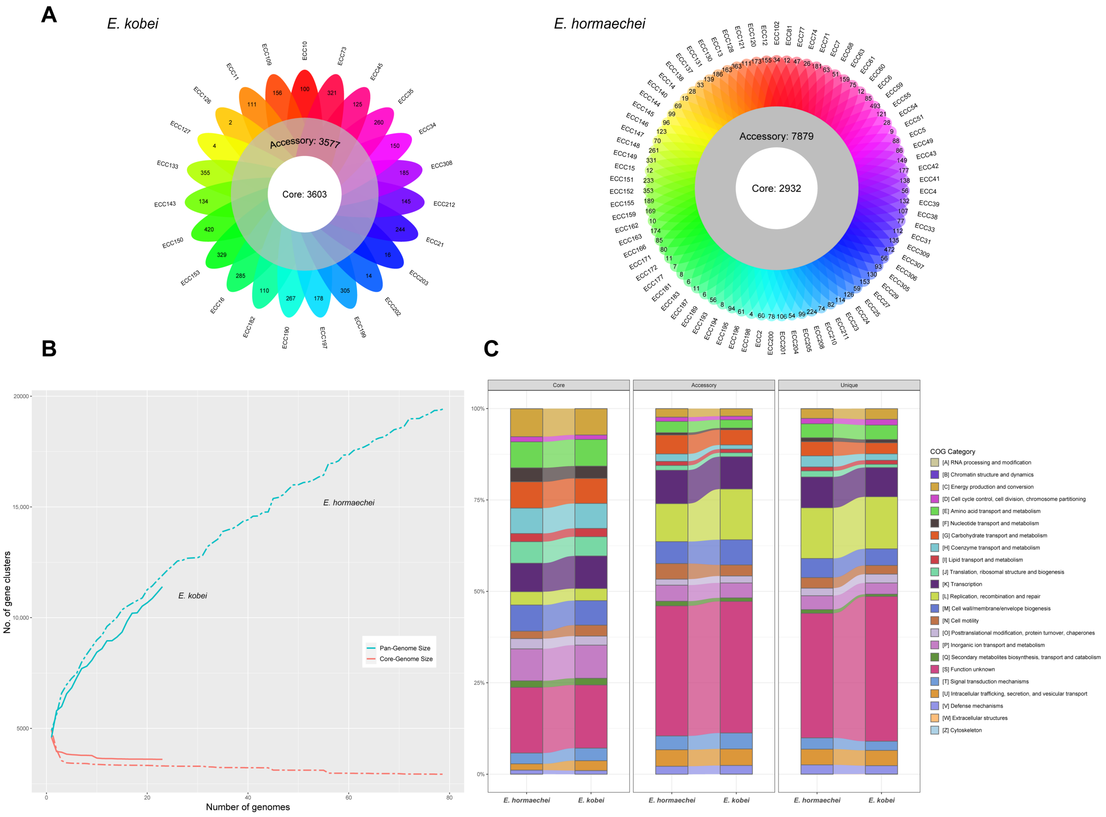

Figure S3. Pan-genome analysis and COG functional annotation of *E. hormaechei* and *E. kobei.* **(A)** Comparative overview of the pan- and core genomes of the two species. **(B)** Gene accumulation curves for pan- (blue) and core genomes (red). **(C)** Distribution of COG functional categories in the core, accessory, and unique genomes of the selected ECC strains.

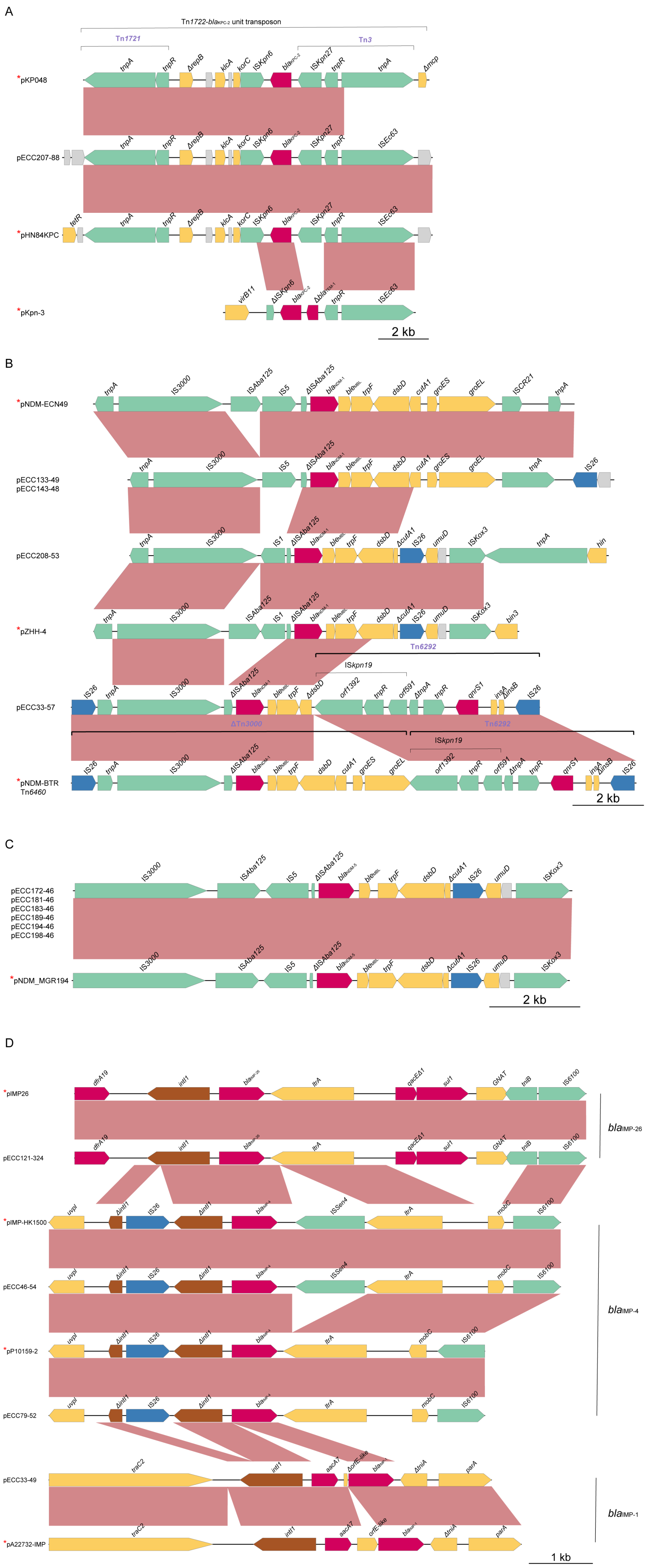

A
Tn1722-blaKPC-2 unit transposon
Tn1721
Tn3
*pKP048
pECC207-88
*pHN84KPC
*pKpn-3
2 kb
B
*pNDM-ECN49
pECC133-49
pECC143-48
pECC208-53
*pZHH-4
Tn6292
ISkpn19
pECC33-57
ΔTn3000
Tn6292
ISkpn19
*pNDM-BTR
Tn6460
2 kb
C
pECC172-46
pECC181-46
pECC183-46
pECC189-46
pECC194-46
pECC198-46
*pNDM_MGR194
2 kb
D
*pIMP26
pECC121-324
blaIMP-26
*pIMP-HK1500
pECC46-54
*pP10159-2
pECC79-52
blaIMP-4
pECC33-49
*pA22732-IMP
blaIMP-1
1 kb

Figure S4. Schematic diagram of the genetic context of carbapenem resistance genes. A, B, C and D, Comparison of the structures of regions carrying $bla_{\text{KPC-2}}$, $bla_{\text{NDM-1}}$, $bla_{\text{NMD-5}}$ and bla_{IMP}. Genes are shown as arrows and colored based on gene function classification. Reference strains are marked with red asterisks.

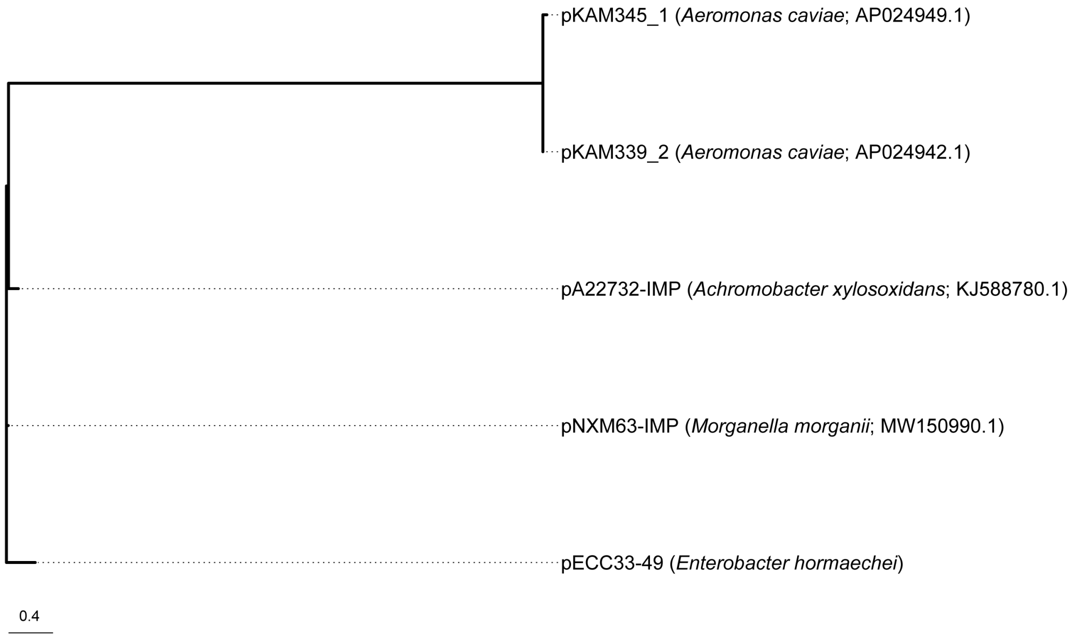

Figure S5. Phylogenetic tree of five IncP1 plasmids. The plasmid pECC33-49 is marked with a black dot.
